# Obstructive Uropathy From a Pelvic Lymphocele After Robotic-Assisted Radical Prostatectomy and Pelvic Lymph Node Dissection: A Case Report

**DOI:** 10.7759/cureus.77980

**Published:** 2025-01-25

**Authors:** Hsuan Ting Kuo, Hsiao Hsien Wang, Chii Cheng Hsieh

**Affiliations:** 1 Department of Urology, Cheng Hsin General Hospital, Taipei, TWN

**Keywords:** lymphocele, obstructive uropathy, pelvic lymph node dissection, prostate cancer, robot-assisted radical prostatectomy, ureteral stent

## Abstract

Lymphocele formation after pelvic lymph node dissection (PLND) in radical prostatectomy (RP) is a well-recognized but often underestimated complication. Although most lymphoceles remain asymptomatic, those that become symptomatic can be troublesome and even life-threatening if venous thromboembolism occurs. We present the case of a 68-year-old man with prostate cancer who underwent an uneventful robotic-assisted RP (RaRP) with bilateral PLND. One month later, he presented with right flank pain, leukocytosis, and acute kidney injury. Imaging revealed large lymphoceles causing right hydronephrosis. A right ureteral stent was placed, immediately relieving symptoms and restoring renal function. At three-month follow-up, a computed tomography (CT) scan demonstrated partial regression of the lymphoceles, and the patient remained symptom-free after stent removal. This case demonstrates alternative management for lymphocele-related complications when it comes to obstructive uropathy using an internal stent to relieve the patient’s symptoms, an option that has not been previously documented in the literature.

## Introduction

Regarding treatment options for prostate cancer, radical prostatectomy (RP) with pelvic lymph node dissection (PLND) is one of the standard choices for patients with intermediate- to high-risk disease [1]. Although PLND itself does not directly improve oncological outcomes, it plays an important role in providing cancer staging and prognostic information [2].

A lymphocele is described as a lymph collection without a clearly defined epithelial lining, caused by disruption of lymphatic channels during procedures such as kidney transplantation, gynecological surgery, and PLND (commonly performed with RP) [3]. Although most lymphoceles are asymptomatic, some develop symptoms, such as fever with chills due to infection, lower abdominal pain, ipsilateral lymphedema, or even thromboembolic events (e.g., deep vein thrombosis or pulmonary embolism) [4].

Post-operative lymphocele formation complicated by obstructive uropathy is rare, with limited references in the literature [5,6]. Herein, we discuss a rare presentation of symptomatic lymphocele causing hydronephrosis following robotic-assisted RP (RaRP) with PLND as well as the use of ureteral stent placement as an effective management option.

## Case presentation

**Figure 1:**
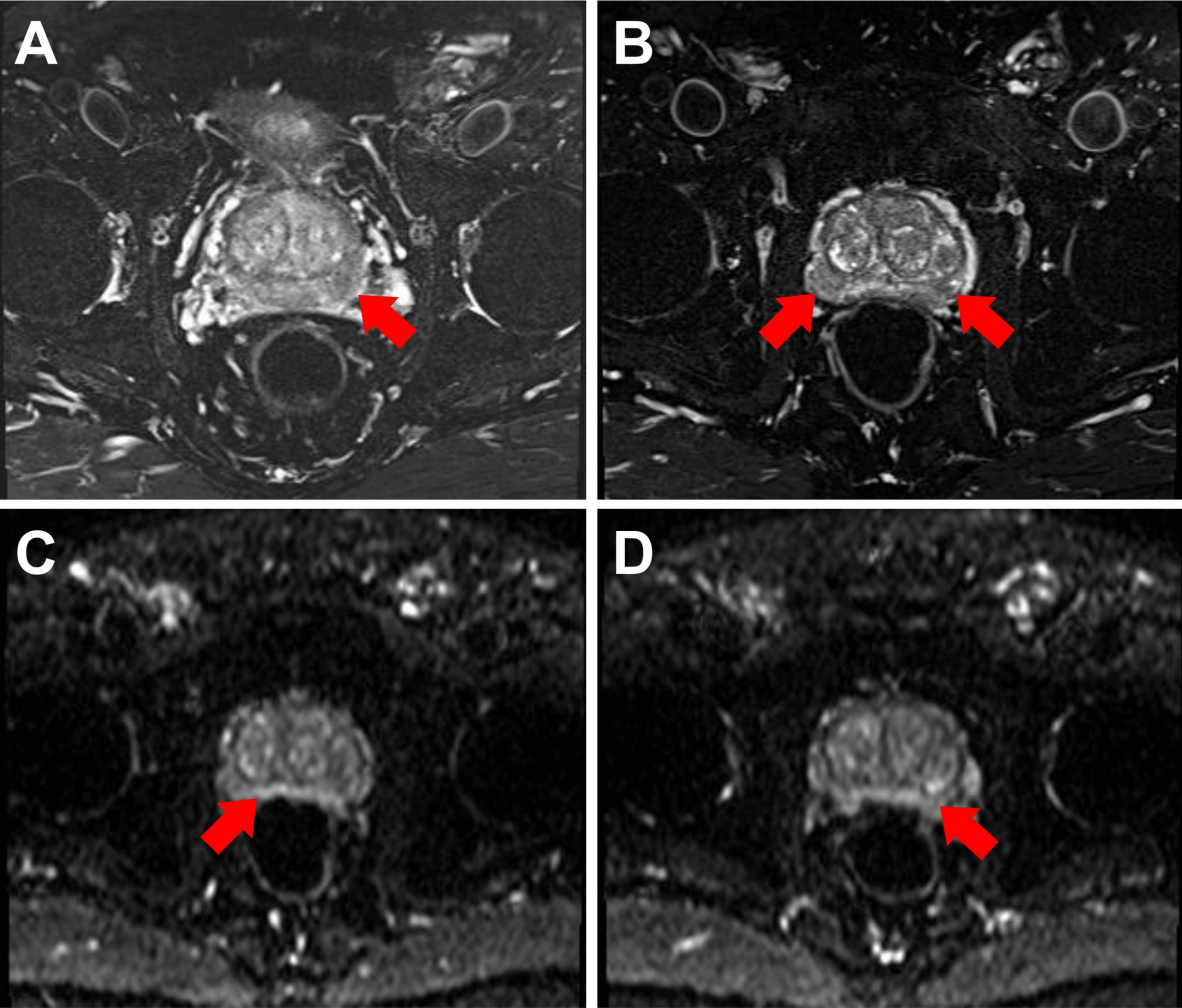
Preoperative multi-parametric MRI of the prostate (A-B) Axial T2-weighted images show hypointense lesions in the bilateral peripheral zones of the prostate (red arrows). (C-D) Diffusion-weighted images show enhanced signals in the bilateral peripheral zones (red arrows). MRI: magnetic resonance imaging

A 68-year-old Taiwanese man without significant systemic medical history presented to our urology outpatient department (OPD) in March 2023 for help due to an elevated prostate-specific antigen (PSA) level noted at an outside clinic. Repeated PSA measurements at our hospital showed an even higher value (14.50 ng/mL). A digital rectal examination showed a moderately enlarged prostate with firm lesions in both lobes. A transrectal ultrasound-guided prostate needle biopsy was performed, and pathology confirmed prostate adenocarcinoma, Gleason score 3+3, Grade Group one, involving two of 12 biopsy cores. A technetium-99m-methylene diphosphonate whole-body bone scan demonstrated no bony metastasis. Multi-parametric magnetic resonance imaging (MRI) (Figure 1) showed a suspicious prostate cancer lesion in the bilateral peripheral zone, with a tentative clinical staging of cT2c N0 M0, stage IIA (according to American Joint Committee on Cancer (AJCC) eighth ), and unfavorable intermediate risk (according to National Comprehensive Cancer Network (NCCN) Prostate Cancer risk stratification) [1,7].

**Figure 2:**
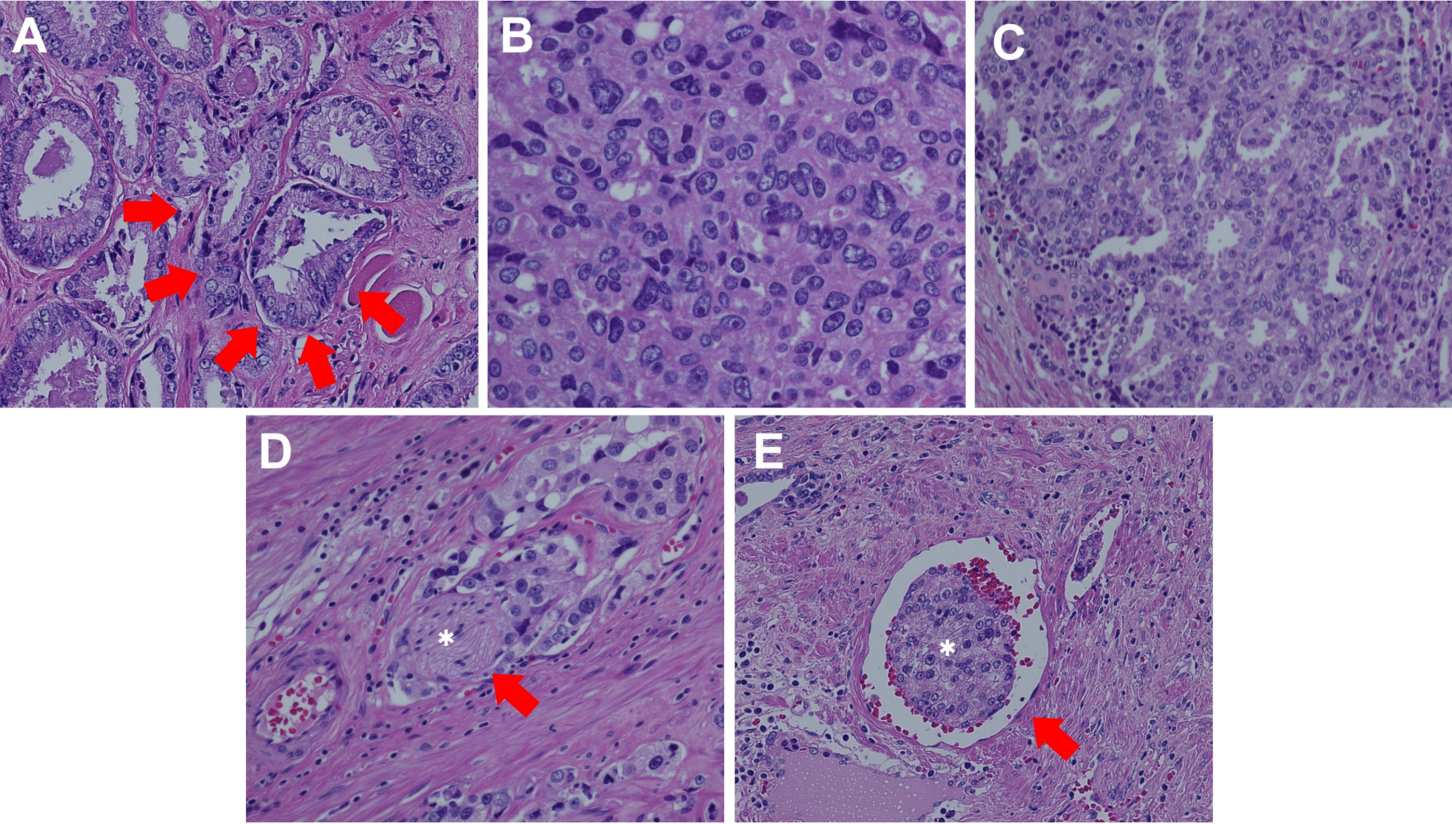
Pathology slides of the prostate (A) "Back-to-back" arrangement of glandular structures (red arrows) indicating Gleason pattern three. (B) Signet ring cells without a glandular appearance indicating Gleason pattern five. (C) The cribriform pattern indicating Gleason pattern four. (D) Nerve (white asterisk) infiltrated by tumor cells (red arrow). (E) Tumor emboli within the lumen of a blood vessel.

We calculated the risk of lymph node involvement using the Memorial Sloan Kettering Cancer Center (MSKCC) nomogram, which showed a low risk (3%) [8]. Following thorough discussion and shared decision-making, the patient underwent RaRP with bilateral PLND (limited template including only obturator lymph nodes) according to NCCN guidelines for prostate cancer. During lymphadenectomy, electrocauterization was used, followed by polymer ligation (Hem-o-lok). The patient had an uneventful postoperative course during hospitalization. The pathology report (Figure 2) of the whole prostate gland and bilateral seminal vesicles revealed prostate acinar adenocarcinoma, Gleason score 3+5, Grade Group four, with no extraprostatic extension or seminal vesicle invasion. However, a positive margin was observed at the distal portion of the left posterior lobe and left apex region (with primary Gleason pattern three only). Lymphovascular and perineural invasion were also noted. A total of 11 nodes were harvested from the right obturator region and four from the left; there was no evidence of metastasis in these lymph nodes. The final pathological stage was pT2 N0 M0, stage IIC.

**Figure 3:**
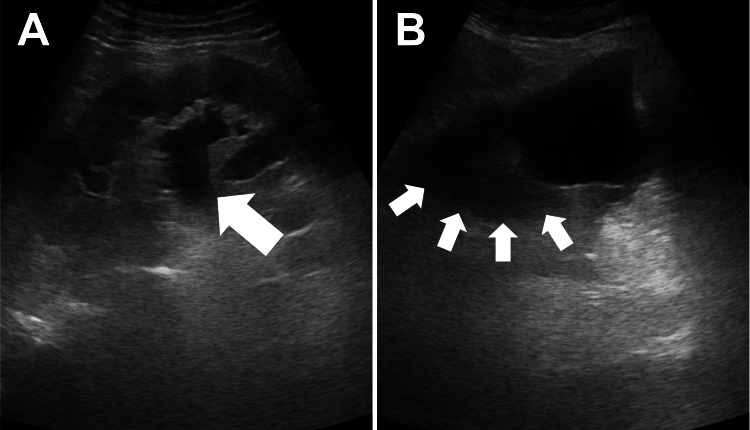
Ultrasound images obtained one month after RaRP with PLND (A) Significant right hydronephrosis was found (white arrow). (B) A hypoechoic peri-vesical area (white arrows) suggests a fluid collection, raising suspicion of a lymphocele. RaRP: robotic-assisted radical prostatectomy; PLND: pelvic lymph node dissection

**Figure 4:**
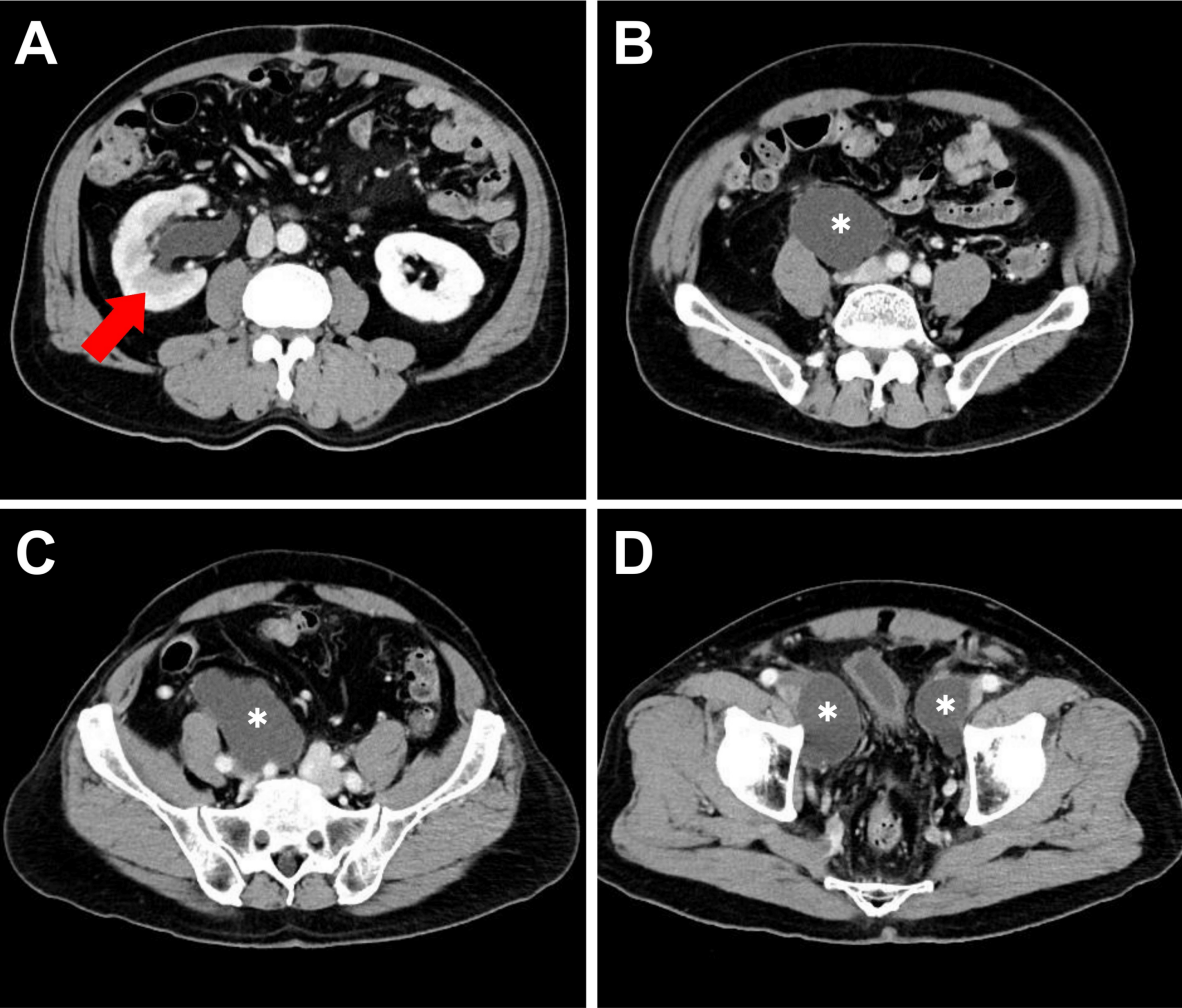
Contrast-enhanced abdominal CT in axial view one month after RaRP with PLND (A) Significant right hydronephrosis (red arrow). (B-D) Multiple lymphoceles (white asterisks) in the right common iliac region (compressing the right ureter), the left external iliac region, and the bilateral peri-vesical area. RaRP: robotic-assisted radical prostatectomy; PLND: pelvic lymph node dissection; CT: computed tomography

**Figure 5:**
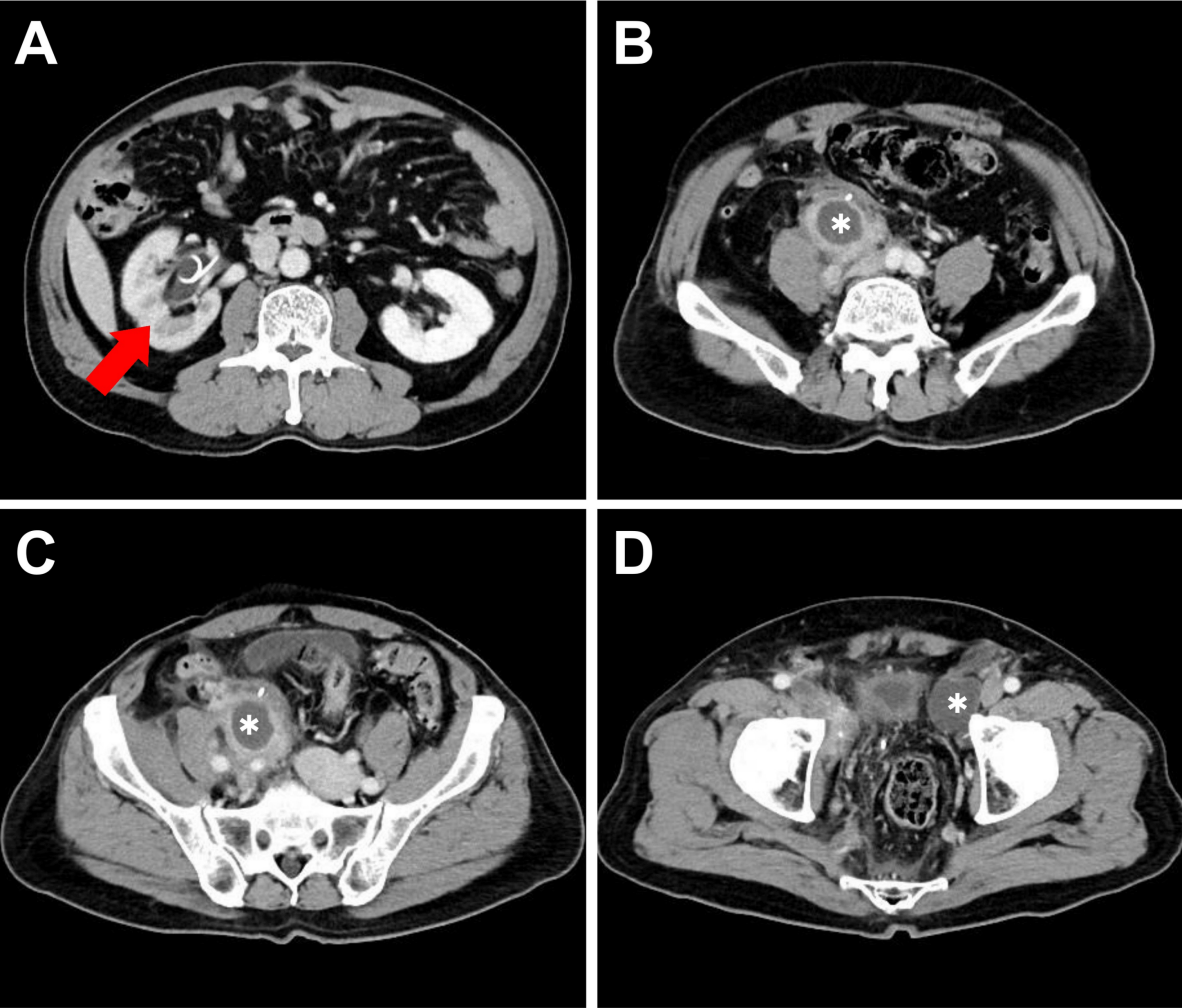
Three-month follow-up contrast-enhanced CT images in axial view after right ureteral stent placement (A) Right hydronephrosis has improved (red arrow). (B-D) The lymphoceles (white asterisks) have partially regressed but become loculated. CT: computed tomography

**Figure 6:**
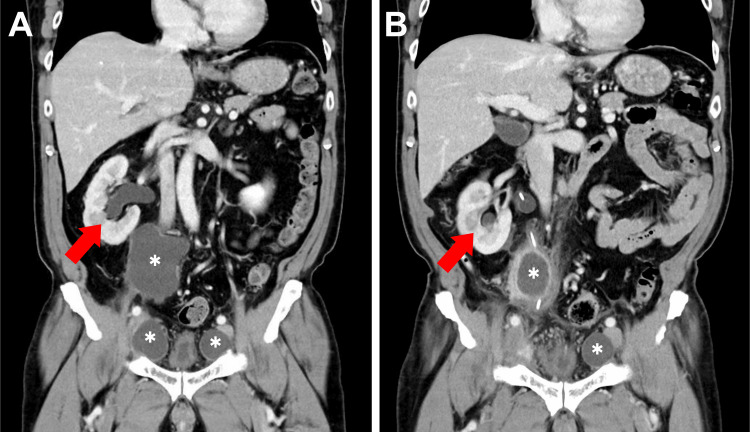
Contrast-enhanced CT images in coronal view demonstrating the regression of lymphoceles and improvement in hydronephrosis over time (A) One month after RaRP with pelvic lymph node dissection, there is significant right hydronephrosis (red arrow) and multiple lymphoceles (white asterisks). (B) Three-month follow-up scan showing a significant reduction in the size of the lymphoceles (white asterisks) and partial resolution of right hydronephrosis (red arrow). CT: computed tomography; RaRP: robotic-assisted radical prostatectomy

One month after the surgery, the patient returned to our OPD with right flank pain for three days. Ultrasonography (Figure 3) showed right hydronephrosis, an intact left kidney, and some hypoechoic material in the peri-vesical region, raising the suspicion of a lymphocele. Blood investigation revealed leukocytosis (white blood cells count=11000 cells/μL) and an elevated serum creatinine level of 1.37 mg/dL (estimated glomerular filtration rate, eGFR=55.1), compared to a baseline of 0.9 mg/dL. Obstructive uropathy-related acute kidney injury was diagnosed according to the Acute Kidney Injury Network (AKIN) and Kidney Disease: Improving Global Outcomes (KDIGO) criteria. Subsequent abdominal computed tomography (CT, Figure 4) revealed a 7.6 cm x 4 cm x 13 cm cystic lesion in the right common iliac region compressing the right ureter, consistent with lymphocele formation causing mild right hydronephrosis. Another cystic lesion measuring 4.9 cm x 3.3 cm was observed in the left external iliac region, also consistent with lymphocele formation. We consulted the interventional radiology service for potential percutaneous drainage; however, the critical anatomical location made the procedure challenging. After discussion with the patient, we performed a cystoscopy with right ureteral stent implantation smoothly. His flank pain was completely relieved with acceptable stent-related discomfort. The creatinine level also fell to baseline (0.92 mg/dL, eGFR=87.2) a month later. A follow-up CT scan three months later (Figures 5, 6) showed partial regression of the bilateral lymphoceles. The ureteral stent was then removed, and the patient remained free of symptoms afterward.

## Discussion

Lymphocele formation following PLND is a well-recognized but often underestimated complication of RP. Incidence rates vary from 9% to 60%, with symptomatic cases in only 2%-5% of patients undergoing either open RP or RaRP [9]. Symptoms include lower abdominal pain, swelling, irritation of adjacent organs, infection, deep vein thrombosis, and, in severe cases, pulmonary embolism [10].

The most definitive way to avoid lymphocele formation after RaRP is to exclude the step of PLND. Several nomograms, including Briganti, Partin, Cagiannos, and the MSKCC nomogram, are used in clinical practice, with published thresholds ranging from 2%-7% [11]. If the risk of PLND-related complications and additional operative time is taken into consideration, PLND can be carried out in patients with favorable intermediate-risk prostate cancer and is recommended for those with unfavorable intermediate-, high-, very-high-risk, or regional prostate cancer according to NCCN guidelines. For patients with very low and low-risk disease, excluding PLND may be an option after appropriate informed consent and discussion of the risks and benefits [1]. Although some patients with positive lymph nodes may be missed, both surgeons and patients should understand that PLND is primarily for pathological staging rather than for therapeutic purposes [2].

Historically, transperitoneal RaRP was believed to have a lower incidence of symptomatic lymphoceles compared with extraperitoneal RaRP because the peritoneum acted as a natural surface for lymph fluid reabsorption. However, one retrospective cohort study showed similar rates and clinical characteristics between the two approaches, possibly due to an overall low event rates that underpowered the study result [12]. The choice of PLND template also affects lymphocele incidence. A recent meta-analysis noted that limited PLND (obturator nodes only) had the lowest rate of lymphocele formation, followed by standard PLND (obturator and external iliac nodes), extended PLND (obturator, external iliac, and internal iliac nodes), and super-extended PLND (obturator, external iliac, internal iliac, common iliac, presacral, and other adjacent lymph nodes) [13].

Differences in surgical approaches used to seal lymphatic vessels during PLND may also influence lymphocele formation. In a randomized trial, Grande et al. compared titanium clips with bipolar coagulation for sealing lymphatic vessels after PLND and found no significant difference in postoperative complications [14].In our case, polymer ligation may function in a similar role to metal clips, despite no literature having mentioned this before. Student et al. introduced a modification in the technique of anchoring the peritoneum to the pubic bone, called "PerFix," and designed a prospective randomized trial to investigate whether this intervention reduces the incidence of lymphoceles. The study showed a significantly decreased incidence of symptomatic lymphoceles but no difference in asymptomatic lymphocele development when conducting PerFix [15]. Three separate meta-analyses also demonstrated a lower incidence of lymphocele formation with peritoneal flap fixation or interposition compared to the conventional approaches [16-18]. In contrast, a sealant or patches have not shown a similar benefit [17].

Most of the lymphoceles are self-limited and can be managed conservatively unless they become symptomatic. The most straightforward management is percutaneous drainage or aspiration by a radiologist under CT- or sonography-guidance. If the lymph leak persists, sclerosing agents such as povidone-iodine, tetracycline, doxycycline, bleomycin, or some novel fibrin sealants can be instilled through the drainage catheter [19]. In refractory cases, laparoscopic or robotic-assisted marsupialization of the lymphocele (in the absence of infection) remains an effective treatment [20,21].

Only two previous case reports have documented hydronephrosis following RaRP [5,6]. Both involved extended PLND during RaRP and subsequent giant pelvic lymphocele formation leading to bilateral hydronephrosis and acute kidney injury, which resolved after receiving ultrasound-guided percutaneous drainage. In our case, the patient underwent RaRP with limited PLND yet developed symptomatic lymphoceles a month later. We performed ureteral stent implantation to the obstructed collecting system, and the symptom was relieved dramatically with regained kidney function. The follow-up image also showed regression of the lymphocele, and the patient remained free of hydronephrosis after stent removal.

## Conclusions

Lymphocele formation is a common complication after RaRP with PLND, though symptomatic cases are relatively rare. Percutaneous drainage remains the standard first-step treatment for symptomatic lymphoceles. In patients with obstructive uropathy, as demonstrated in this case, endoscopic ureteral stenting can be a feasible alternative, particularly when percutaneous drainage is technically challenging due to anatomical concerns, in patients on anticoagulants, or in those who prefer a less invasive procedure.
